# Functional Dyspepsia Among Nurses Working Rotating Shifts: Its Prevalence and Associations With Sleep Quality and Stress

**DOI:** 10.7759/cureus.101887

**Published:** 2026-01-20

**Authors:** Rishit Gupta, Arihant Senthil, Smita Nath, Rajnish K Avasthi, Shrey Chopra, Sukul Khanna

**Affiliations:** 1 Internal Medicine, University College of Medical Sciences, New Delhi, IND

**Keywords:** dyspepsia, epigastric pain syndrome, functional dyspepsia, nurses, perceived stress scale 10, pittsburgh sleep quality index, postprandial distress syndrome, shift work, sleep quality, stress

## Abstract

Background and objective

Functional dyspepsia (FD) is one of the most common functional gastrointestinal disorders that frequently impairs quality of life. Among healthcare professionals, shift work may heighten the risk of developing this condition, likely through its impact on stress levels and sleep quality. This study aimed to determine the prevalence of FD among nurses working rotating shifts in India and to examine its relationship with sleep quality and perceived stress.

Methods

A cross-sectional study was conducted at a tertiary care hospital in New Delhi (January 2022-March 2023). The participating nurses (n=100) completed standardized questionnaires, including the Rome IV criteria for FD diagnosis, Pittsburgh Sleep Quality Index (PSQI), and Perceived Stress Scale-10 (PSS-10). Statistical analyses included chi-square tests to evaluate associations.

Results

The prevalence of FD in the cohort was 31%, with 12% reporting postprandial distress syndrome (PDS), 10% epigastric pain syndrome (EPS), and 9% both. Poor sleep quality (PSQI ≥5) was observed in 53% of participants, while 50% reported high/very high stress levels as per PSS-10. Significant correlations emerged between FD and poor sleep (74.2% vs. 43.5% in non-FD, p=0.003) and high stress (83.8% vs. 34.7%, p<0.001). Similar trends were observed for PDS and EPS individually. No gender-based differences in FD prevalence were identified.

Conclusions

Rotating shift work is associated with a high FD prevalence (31%) among nurses, surpassing both the estimated prevalence in the general Indian population (30.4%) and figures reported in other countries. Poor sleep quality and elevated stress levels were strongly linked to FD, highlighting occupational health risks in healthcare shift workers. These findings underscore the need for workplace interventions targeting sleep hygiene and stress management. Future longitudinal studies with control groups are warranted to establish causal relationships and devise preventive strategies.

## Introduction

Dyspepsia is an umbrella term for chronic or recurrent upper abdominal symptoms such as pain, discomfort, postprandial fullness, or early satiety. Studies have shown that about 80% of individuals with dyspepsia show no abnormalities on diagnostic evaluations, including upper gastrointestinal (GI) endoscopy. Most patients with symptoms of dyspepsia can, therefore, be assumed to have functional dyspepsia (FD), i.e., dyspepsia without any apparent organic cause [1,2,3]. Cross-sectional surveys applying symptom-based criteria for assigning a diagnosis of dyspepsia without an endoscopy thus offer a close approximation of FD prevalence [2].

The exact pathogenesis of FD remains uncertain. Multiple complex pathophysiological mechanisms have been proposed, many of which may overlap and contribute to the development and progression of the disorder: altered GI motility and sensory dysfunction, immune-mediated GI inflammation, changes in the gut microbiome, and improper gut-brain signaling. Evidence also suggests that among a majority of healthcare seekers, psychiatric conditions like anxiety and depression precede various functional GI disorders (FGIDs), including FD, thereby lending credence to a possible contributory psychopathological mechanism [4].

FD is a fairly common condition, with prevalence rates ranging from 5 to 40% in various studies. This broad range likely reflects geographical differences and inconsistencies in the diagnostic criteria used to define FD. The prevalence of FD based on the Rome diagnostic criteria is typically 6-14% higher in Western countries compared to Eastern countries [5]. Shah et al. in their study found that the prevalence of dyspepsia in urban India can be as high as 30.4% [6]. In industrialized countries, as much as 75% of the workforce is estimated to engage in shift or night work [7].

Shift work is linked to a higher risk of obesity, diabetes, cardiovascular disease, malignancies, and immune system disorders [8]. Additionally, irregular shift work is strongly associated with increased GI disturbances or FGIDs [9]. Due to the continuous demands associated with human health and disease management, healthcare facilities must operate 24/7, making shift work an essential component of the healthcare system. A study in South Korea reported that nurses working rotating shifts had a 19.7% prevalence of FD [10]. The primary aim of our study is to determine the prevalence of FD in nurses working in rotating shifts.

Two significant impacts of shift work are a disturbed sleep cycle and a higher level of emotional stress. The irregular sleep cycle of shift workers has been associated with disturbances in the rhythmic secretion of melatonin and cortisol [11]. In a study by Klupińska et al., regular intake of melatonin was found to be associated with significant symptom reduction in patients with FD [12]. This suggests that disruption in the normal circadian rhythm, a primary component of which is melatonin secretion, could be a significant contributor to the development of FD. Moreover, emotional stress has been linked to FD development in younger patients [13]. The secondary objective of this study is to appraise the relationship between sleep quality and emotional stress with the development of FD.

## Materials and methods

Study design and setting

This was a cross-sectional hospital-based study that was conducted between January 2022 and March 2023 at the University College of Medical Sciences and GTB Hospital, New Delhi, which is a tertiary care hospital.

Study population and eligibility criteria

Hospital nurses were invited to complete standardized, self-administered questionnaires following a detailed explanation of the process and after obtaining written informed consent. Participation was completely voluntary and uncompensated. Out of the 124 nurses who initially volunteered, 24 were excluded based on the inclusion and exclusion criteria.

Inclusion Criteria

The inclusion criteria were as follows: (1) nurses aged 18-60 years who gave consent, and (2) participants with at least one year of service in the healthcare system.

Exclusion Criteria

The exclusion criteria were as follows: (1) lactating or pregnant women; (2) individuals with a history of inflammatory bowel disease; (3) those who had undergone previous gastrointestinal surgery; and (4) participants with a prior diagnosis of cancer, acid peptic disease, or any history of smoking or alcohol consumption.

Survey questionnaires

Four distinct sets of questionnaires, comprising a total of 44 questions, were employed in this study. First, a general pro forma was used to collect demographic data and ensure adherence to the inclusion and exclusion criteria. Next, the diagnosis of FD was based on the Rome IV criteria and was assessed using the Rome IV questionnaire [14]. Sleep quality was evaluated using the Pittsburgh Sleep Quality Index (PSQI), a validated instrument for assessing sleep in adults. The PSQI differentiates between “good” and “poor” sleep quality by analyzing seven domains: subjective sleep quality, sleep latency, sleep duration, habitual sleep efficiency, sleep disturbances, use of sleep medications, and daytime dysfunction over the preceding month. A total score of 5 or higher signifies poor sleep quality [15]. In addition, the Perceived Stress Scale-10 (PSS-10) was administered to provide an initial assessment of stress levels. The Perceived Stress Scale-10 (PSS-10) offers a validated framework for categorizing stress into five distinct groups. The 10-item questionnaire assigns scores of 0-7 to "very low health concern," 8-11 to "low health concern," 12-15 to "average health concern," 16-20 to "high health concern," and 21 or above to "very high health concern" [16]. This detailed stratification allows for a good understanding of the severity of stress in the study population.

Statistical analysis

The data collected from the above-mentioned questionnaires were entered into an MS Excel sheet and then transferred to SPSS Statistics software 20.0 (IBM Corp., Armonk, NY). Scoring and categorization were done as mentioned in the questionnaire. Descriptive tables were generated, and the chi-square test was applied to find associations, if any, between different parameters. The statistical significance level was set at 5%.

Ethical considerations

The study was conducted after obtaining approval from the Institutional Ethics Committee-Human Research (IECHR), in accordance with the Declaration of Helsinki at the University College of Medical Sciences and GTB Hospital.

Informed consent

Hospital nurses were asked to fill out standardized, self-reported questionnaires after receiving a thorough explanation of the study and providing written informed consent. Their involvement was completely voluntary, and no incentives were offered.

## Results

Our study was conducted among nurses engaged in rotating shift work at GTB Hospital and involved a total of 100 participants. The participants were comprised of 83% females (n=83) and 17% males (n=17).

Prevalence of FD and its subtypes

Based on their responses, 31% (n=31) of patients had FD, and 69% (n=69) did not. The Rome IV questionnaire classifies FD into two subcategories, postprandial distress syndrome (PDS) and epigastric pain syndrome (EPS). Among all patients, 12% (n=12) had PDS only, 10% (n=10) had EPS only, and 9% (n=9) had both PDS and EPS. Accordingly, when including overlapping cases, 21% of participants (n=21) had PDS (either alone or with concurrent EPS), and 19% (n=19) had EPS (either alone or with concurrent PDS). These percentages are not mutually exclusive, as patients who meet the criteria for both subtypes are counted in both groups.

Assessment of sleep quality and stress amongst participants

Based on the results of the PSQI questionnaire, 53% (n=53) of the patients had poor sleep quality, while 47% (n=47) did not. Perceived stress was measured using the PSS-10 questionnaire: 24% (n=24) of the patients were categorized under ‘very high health concern’, 26% (n=26) under ‘high health concern’, 27% (n=27) under ‘average health concern’, 16% (n=16) under ‘low health concern’, and 7% (n=7) under ‘very low health concern’.

Correlation of biological sex with various parameters

Table 1 shows the distribution of FD by age group and sex among our participants.

**Table 1:** Distribution of FD by age group and sex FD: functional dyspepsia

Demographic	FD	No FD
Age ≤35 years (n=51)	13 (25.5%)	38 (74.5%)
Age >35 years (n=49)	18 (36.7%)	31 (63.3%)
Female (83)	26 (31.3%)	57 (68.7%)
Male (17)	5 (29.4%)	12 (70.6%)

Of note, 83% of the participants (n=83) were female, and 17% (n=17) were male in our study. No statistically significant correlation was found between biological sex and any of the measured parameters. Out of the 31 patients who had FD, 26 were female (p=0.225). Twenty-one patients had PDS, and 18 of these were female (p=0.389). 19 patients had EPS, and 15 of these were female (p=0.490). Among females, about half (50.6%) had poor sleep quality, and among males, a majority (64.7%) had poor sleep quality. Based on responses to the PSS-10 questionnaire, perceived stress represented a high or very high health concern for 53% of female participants and 35.2% of male participants.

Correlation of perceived stress with FD

**Figure 1:**
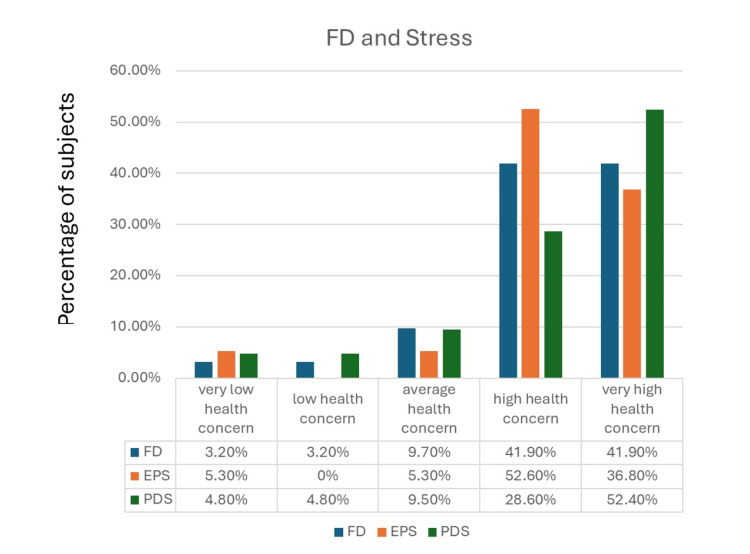
Association between FD and stress FD: functional dyspepsia; EPS: epigastric pain syndrome; PDS: postprandial distress syndrome

The presence of FD was strongly associated with higher perceived stress. Figure 1 illustrates the relationship of FD and its subtypes with stress.

Overall, 83.8% of patients with FD fell into the high or very high stress category (p=0.000), compared with only 34.7% of those without FD. A similar pattern was observed in both subtypes. Among patients with PDS, 81% reported high or very high stress (p=0.000), versus 41.8% of PDS-negative individuals. Likewise, 89.4% of patients with EPS were categorized as having high or very high stress according to PSS-10 (p=0.001), compared with 40.8% of those without EPS.

Correlation of sleep quality with FD

**Figure 2:**
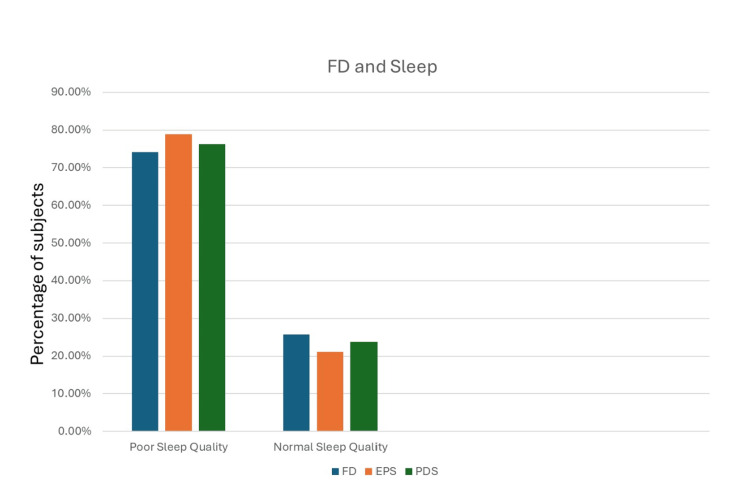
Association between FD and sleep quality FD: functional dyspepsia; EPS: epigastric pain syndrome; PDS: postprandial distress syndrome

FD was also significantly correlated with poor sleep quality. Figure 2 demonstrates the relationship of FD and its subtypes with sleep quality.

Among individuals with FD, 74.2% reported poor sleep quality (PSQI score ≥5; p=0.003), compared with 43.5% of those without FD.

Comparable findings emerged when FD was stratified by subtypes. Poor sleep was present in 76.2% of patients with PDS (p=0.001) compared with 46.8% of those without PDS. Similarly, 78.9% of EPS-positive patients had poor sleep quality (p=0.008), versus 46.9% of EPS-negative individuals. Table 2 summarizes these findings.

**Table 2:** Sleep quality and perceived stress stratified by FD subtype FD: functional dyspepsia

Group	Poor sleep quality (%)	P-value	High/very high stress (%)	P-value
FD (n=31)	74.2	0.003	83.8	0.000
PDS (n=21)	76.2	0.01	81.0	0.000
EPS (n=19)	78.9	0.008	89.4	0.001

## Discussion

To our knowledge, this is the first study to use the Rome IV criteria for calculating the prevalence of FD in the Indian context. Rome IV divides FD into two subcategories, PDS and EPS. Patients may be assigned one or both of these diagnoses based on which symptoms predominate in their responses. In this cross-sectional questionnaire-based study, we found a 31% prevalence of FD among our subjects, all of whom were nurses working in rotating shifts; 12% of respondents had only PDS, 10% had only EPS, and 9% had both. In comparison, Shah et al. [6] reported a dyspepsia prevalence of 30.4% in the general urban Indian population, indicating that the prevalence of FD in our study group is comparatively higher. Similar studies conducted in South Korea reported the prevalence of FD among nurses working rotating shifts to be 19.7% [10] and 22.2% [17], respectively. Nagarethinam et al. reported that 19% of healthcare professionals in a tertiary care center in Australia were diagnosed with FD [18]. Several studies have also shown that non-day shift workers experience more GI symptoms than those on regular day shifts [9,19,20], with comparable patterns observed for functional bowel disorders [21] and IBS [22]. This suggests a possible link between shift work and FD.

According to our study, neither biological sex significantly correlated with FD incidence. Various studies have found that females are at a relatively higher risk of developing FD [2,23]. However, our study found no correlation between biological sex and the risk of developing FD.

Correlation of FD with sleep quality

Shift work is known to cause disturbances in the sleep cycle as well as reduced quality of sleep, and various studies have shown these to be significant contributors to the development of GI disturbances. In a study by Torsvall et al., the average duration of sleep following a night shift was reduced by more than two hours compared with sleep after an afternoon shift. This reduction predominantly affected REM (rapid eye movement) and Stage 2 NREM (non-rapid eye movement) sleep, both of which play important roles in long-term memory consolidation [24]. Additionally, 28% of night-shift patients also reported adding a spontaneous afternoon nap to their main sleep after their night shift [25]. These findings suggest that shift work causes significant alterations in sleep quality, duration, and pattern. 

Roehrs et al. showed that modest reductions in sleep, and, specifically, REM sleep deprivation, had a somatic hyperalgesic effect. Their findings also indicated that the effects of sleep loss accumulated over multiple nights of sleep deprivation and further contributed to hyperalgesia [26]. Possible pathophysiological mechanisms that might explain this finding include decreased cholinergic activity, a finding observed in rats deprived of REM sleep [27], and depletion of serotonin and its metabolite (5-HIAA) [28,29]. An important concept in sleep physiology is that of the circadian rhythm, colloquially referred to as the 24-hour biological clock. Reduced night sleep time and compensatory daytime sleep are associated with disturbances in hormonal secretions, an important component of the circadian rhythm [11].

In addition, the circadian rhythm has been shown to affect gut visceral sensitivity [30]. This is further evidenced by the findings of Nojkov et al., who interestingly concluded that increased IBS prevalence in rotating shift workers was a consequence of circadian rhythm disruption, rather than poor sleep quality [22]. In our study, poor sleep quality was significantly associated with a higher risk of FD. Out of all the participants who had FD, about three-quarters had poor sleep. This is consistent with a study done in South Korea, which also shows a significant correlation between poor sleep quality and FD [17]. We believe that both poor sleep quality as well as a disturbed circadian rhythm in nurses working in rotating shifts might contribute to the increased prevalence of FD in this population.

Correlation of FD with perceived stress

Shift work has been associated with higher stress levels, and higher perceived stress has, in turn, been linked to an increased risk of GI morbidity. Night shift work has been associated with increased occupational stress among nurses [31]. Furthermore, among nurses, psychosocial problems are more common amongst those working rotating shifts than those working during the day [32]. The gut-brain axis is an emerging concept that outlines bidirectional communication between cognitive and emotional centers of the brain and the enteric nervous system. The central nervous system has a definite role in peripheral intestinal functioning and may play a role in various GI pathologies [33]. Improper gut-brain signaling has been implicated in the development of various FGIDs. This might, in part, explain a possible psychopathological mechanism behind FGID development.

According to our findings, a substantial majority of patients with FD (83.8%) exhibited stress levels indicative of significant health concern. In contrast, only 43.5% of patients without FD demonstrated comparable stress levels. These findings align with other studies that have analyzed the correlation between GI problems and psychological factors. Higher perceived levels of stress have been associated with an increased incidence of GI symptoms in healthcare professionals [18]. Koh et al. concluded that significant psychosocial distress is an independent risk factor for the development of FD [10]. According to a population-based survey conducted in Sweden, participants with anxiety had higher odds of developing FD compared to those without FD [34].

Our findings suggest that perceived stress and poor sleep quality are strongly associated with the presence of functional dyspepsia among nurses engaged in rotating shift work. While these associations are statistically significant and consistent with prior literature, the cross-sectional nature of our study precludes causal inference. It is therefore more appropriate to interpret these results as correlational rather than causal. Future longitudinal or interventional studies with larger sample sizes and multivariate analyses are warranted to clarify the independent contributions of stress, sleep, and other occupational factors to FD risk.

Limitations

This study has several limitations. Being single-center and cross-sectional, its findings may not be generalizable and cannot establish causality. The absence of a control group and the potential influence of unmeasured confounders limit internal validity. Diagnostic accuracy may also have been affected by the lack of endoscopic confirmation, as FD was defined solely by Rome IV symptom criteria. In addition, the modest response rate raises the possibility of selection bias, as nurses experiencing symptoms may have been more likely to participate. Finally, external stressors related to variable work schedules could not be fully accounted for, which may have influenced the observed associations.

## Conclusions

This study found a 31% prevalence of FD among nurses working rotating shifts, which is higher than rates reported in many international cohorts and slightly exceeds estimates for the general Indian population. Poor sleep quality was significantly associated with FD, with the majority of affected nurses reporting suboptimal sleep, suggesting that circadian rhythm disruption may play a key role in symptom development. Additionally, perceived stress was notably greater in nurses with FD compared to those without, supporting the involvement of stress-related mechanisms in FD pathophysiology. These findings highlight the occupational health risks faced by nurses engaged in shift work, underscoring the need for institutional policies and workplace interventions that promote sleep hygiene, stress management, and mental health support. Identifying at-risk individuals through screening and providing targeted interventions may help alleviate the burden of FD and enhance the well-being and productivity of healthcare professionals. Future longitudinal studies involving larger and more diverse populations, the inclusion of control groups, and the use of objective diagnostic methods such as endoscopy are needed to clarify causal relationships and improve preventive strategies. Ultimately, addressing modifiable factors such as sleep and stress in this vulnerable workforce could contribute not only to better GI health but also to enhanced quality of life and occupational performance.

## Appendices

Questionnaire

Functional Dyspepsia in Nurses working in rotating shifts

This is a consent form for nurses between 18-60 years working in rotating shifts for more than 1 year. We invite you to participate in the study- "Functional Dyspepsia in nurses working in rotating shifts". 

Who is conducting this study?

Rishit Gupta, 3rd year medical student, and Arihant Senthil, 3rd year medical student, under the supervision and guidance of Professor and Head Dr. R. Avasthi of the Department of Medicine and Associate Professor Dr. Smita Nath of the Department of Medicine, University College of Medical Sciences, Delhi.

Why?

To find the prevalence of functional dyspepsia in rotating shift workers, like nurses.

Benefits and risks: The study provides the prevalence of functional dyspepsia in nurses working in rotating shifts. The fact that healthcare professionals in India work in frequently rotating shifts makes this study very important for appreciating the problems that can occur due to this work schedule. 

Ethical consideration: The information that is collected from this study will be kept confidential. De-identified data will be used during analysis and presentation. 

Your participation in this research is entirely voluntary. 

We request that you fill this form with full sincerity.

In case of queries before, during, or after completing the questionnaire, you can contact Rishit Gupta @8800496812 

1. I have fully read and understood the information given above. I consent to * 

voluntarily participate in the study. 

Mark only one oval. 

Yes 

No 

General Proforma 

2. Mobile number * 

3. Age* 

4. Gender * 

Mark only one oval. 

Male 

Female 

Transgender 

5. Are you currently pregnant? * 

Mark only one oval. 

Yes 

No 

6. Are you currently breastfeeding? * 

Mark only one oval. 

Yes 

No 

7. Any history of previously diagnosed inflammatory bowel disease (ulcerative colitis * or Crohn’s disease?) 

Mark only one oval. 

Yes 

No 

8. Did you undergo any surgery involving the gastrointestinal tract in the past? *

Mark only one oval. 

Yes 

No 

9. Any history of previously diagnosed cancer of any type? + * 

Mark only one oval. 

Yes 

No 

10. Any history of previously diagnosed acid peptic disease? * 

Mark only one oval. 

Yes 

No 

11. Any history of intake of the following? * 

Check all that apply. 

Smoking 

Alcohol >7 drinks/week 

Alcohol >14 drinks/week 

None 

The following section will assess for gastrointestinal conditions

12. In the last 3 months, how often did you feel so full after a regular-sized meal (the * amount you normally eat) that it interfered with your usual activities?) 

Mark only one oval. 

Less than one day a month 

One day a month 

2-3 days a month 

Once a week 

2-3 days a week 

Most days 

Everyday 

Multiple times per day or all the time 

13. Has it been 6 months or longer since you started having these episodes of * fullness after meals that was severe enough to interfere with your usual activities? 

Mark only one oval. 

Yes 

No 

14. In the last 3 months, how often were you unable to finish a regular-sized meal * because you felt too full? 

Mark only one oval. 

Less than one day a month 

One day a month 

2-3 days a month 

Once a week 

2-3 days a week 

Most days 

Everyday 

Multiple times per day or all the time 

15. Has it been 6 months or longer since you started having these episodes of feeling * too full to finish regular-sized meals? 

Mark only one oval. 

Yes 

No 

16. In the last 3 months, how often did you have pain or burning in the middle part of * your upper abdomen (above your belly button but not in your chest), that was so severe that it interfered with your usual activities? 

Mark only one oval. 

Less than one day a month 

One day a month 

2-3 days a month 

Once a week 

2-3 days a week 

Most days 

Everyday 

Multiple times per day or all the time 

17. Has it been 6 months or longer since you started having this pain or burning in the * middle part of your upper abdomen? 

Mark only one oval. 

Yes 

No 

The following section will assess Mental health and Sleep quality 

18. In the last month, how often have you been upset because of something that * happened unexpectedly? 

Mark only one oval. 

Never 

Almost never 

Sometimes

Fairly often 

Very often 

19. In the last month, how often have you felt that you were unable to control the * important things in your life? 

Mark only one oval. 

Never 

Almost never 

Sometimes 

Fairly often 

Very often 

20. In the last month, how often have you felt nervous and “stressed”? * 

Mark only one oval. 

Never 

Almost never 

Sometimes 

Fairly often 

Very often 

21. In the last month, how often have you felt confident about your ability to handle * your personal problems? 

Mark only one oval. 

Never 

Almost never 

Sometimes 

Fairly often 

Very often 

22. In the last month, how often have you felt that things were going your way? * 

Mark only one oval. 

Never 

Almost never 

Sometimes 

Fairly often 

Very often 

23. In the last month, how often have you found that you could not cope with all the * things that you had to do? 

Mark only one oval. 

Never 

Almost never 

Sometimes 

Fairly often 

Very often 

24. In the last month, how often have you been able to control irritations in your life? * 

Mark only one oval. 

Never 

Almost never 

Sometimes 

Fairly often 

Very often 

25. In the last month, how often have you felt that you were on top of things? * 

Mark only one oval. 

Never 

Almost never 

Sometimes 

Fairly often 

Very often 

26. In the last month, how often have you been angered because of things that were * outside of your control? 

Mark only one oval. 

Never 

Almost never 

Sometimes 

Fairly often 

Very often 

27. In the last month, how often have you felt difficulties were piling up so high that * you could not overcome them? 

Mark only one oval. 

Never 

Almost never 

Sometimes 

Fairly often 

Very often 

28. How long (in minutes) has it taken you to fall asleep each night? * 

Mark only one oval. 

<15 min 

16-30 min 

31-60 min 

>60 min 

29. How many hours of actual sleep did you get at night? * 

30. How many hours were you in bed? * 

During the past month, how often have you had trouble sleeping because you:

Kindly answer the next 10 questions with respect to the above heading. 

31. Cannot get to sleep within 30 minutes * 

Mark only one oval. 

Not during the past month 

Less than once a week 

Once or twice a week 

Three or more times a week 

32. Wake up in the middle of the night or early morning * 

Mark only one oval. 

Not during the past month 

Less than once a week 

Once or twice a week 

Three or more times a week 

33. Have to get up to use the bathroom * 

Mark only one oval. 

Not during the past month 

Less than once a week 

Once or twice a week 

Three or more times a week 

34. Cannot breathe comfortably * 

Mark only one oval. 

Not during the past month 

Less than once a week 

Once or twice a week 

Three or more times a week 

35. Cough or snore loudly * 

Mark only one oval. 

Not during the past month 

Less than once a week 

Once or twice a week 

Three or more times a week 

36. Feel too cold * 

Mark only one oval. 

Not during the past month 

Less than once a week 

Once or twice a week 

Three or more times a week 

37. Feel too hot * 

Mark only one oval. 

Not during the past month 

Less than once a week 

Once or twice a week 

Three or more times a week 

38. Have bad dreams * 

Mark only one oval. 

Not during the past month 

Less than once a week 

Once or twice a week 

Three or more times a week 

39. Have pain * 

Mark only one oval. 

Not during the past month 

Less than once a week 

Once or twice a week 

Three or more times a week 

40. Other reasons, please describe, including how often you have had trouble * sleeping because of this reason 

Mark only one oval. 

Not during the past month 

Less than once a week 

Once or twice a week 

Three or more times a week 

None 

Other: 

41. During the past month, how often have you taken medicine (prescribed or “over * the counter”) to help you sleep? 

Mark only one oval. 

Not during the past month 

Less than once a week 

Once or twice a week 

Three or more times a week 

42. During the past month, how often have you had trouble staying awake while driving, eating meals, or engaging in social activity? 

Mark only one oval. 

Not during the past month 

Less than once a week 

Once or twice a week 

Three or more times a week 

43. During the past month, how much of a problem has it been for you to keep up the enthusiasm to get things done? 

Mark only one oval. 

Not during the past month 

Less than once a week 

Once or twice a week 

Three or more times a week 

44. During the past month, how would you rate your sleep quality overall? * 

Mark only one oval. 

Very good 

Fairly good 

Fairly bad 

Very bad 
